# Machine Learning Based Network Analysis Determined Clinically Relevant miRNAs in Breast Cancer

**DOI:** 10.3389/fgene.2020.615864

**Published:** 2020-11-12

**Authors:** Min Qiu, Qin Fu, Chunjie Jiang, Da Liu

**Affiliations:** ^1^Department of Orthopedics, Shengjing Hospital of China Medical University, Shenyang, China; ^2^Institute for Diabetes, Obesity, and Metabolism, Perelman School of Medicine, University of Pennsylvania, Philadelphia, PA, United States; ^3^Division of Endocrinology, Diabetes and Metabolism, Department of Medicine, Perelman School of Medicine, University of Pennsylvania, Philadelphia, PA, United States; ^4^Department of Obstetrics and Gynecology, Shengjing Hospital of China Medical University, Shenyang, China

**Keywords:** breast cancer, miRNA, SVM classifier, immune infiltration, drug response

## Abstract

**Background and Aims:**

MicroRNAs (miRNAs) have been shown to play important roles in many cancers, including breast cancer. The majority of previous studies employed network analysis to identify key miRNAs in cancer progression. However, most of dysregulated miRNA networks were constructed based on the expression variation of miRNAs and target genes.

**Methods:**

The relations between miRNAs and target genes were computed by Spearman correlation separately in breast cancer and normal breast samples. We calculated dysregulated scores based on the dysregulation of miRNA-mRNA regulatory relations. A dysregulated miRNA target network (DMTN) was constructed from the miRNA-mRNA pairs with significant dysregulated scores. SVM classifier was employed to predict breast cancer risk miRNAs from the DMTN. Hypermetric test was utilized to calculate the significance of overlap between different gene sets. Pearson correlation was used to evaluate associations between miRNAs/genes and drug response.

**Results:**

The DMTN comprised 511 miRNAs and was similar to common biological networks. Based on miRNAs and target genes in DMTN, we predicted 90 breast cancer risk miRNAs by using SVM classifier. Predicted risk miRNAs and one-step neighbor genes were significantly overlapping with differential miRNAs, cancer-related and housekeeping genes in breast cancer. These risk miRNAs were involved in many cancer-related and immune-related processes. In addition, most risk miRNAs were able to predict survival of breast cancer patients. More interestingly, some risk miRNAs and one-step neighbor genes were remarkably associated with immune cell infiltration. For example, high expression of hsa-miR-155 indicates high abundance of activated CD4+ T cells but low level of M2 macrophage infiltration. Furthermore, we identified 588 miRNA-drug and 3,146 gene-drug pairs, wherein the expression level of miRNAs/genes could indicate the sensitivity of cancer cells to anti-cancer drugs.

**Conclusion:**

We predicted 90 breast cancer risk miRNAs based on proposed DMTN by using SVM classifier. Predicted risk miRNAs are biologically and clinically relevant in breast cancer. Risk miRNAs and one-step neighbor genes could serve as biomarkers for immune cell infiltration and anti-cancer drug response, which sheds lights on immunotherapy or targeted therapy for patients with breast cancer.

## Introduction

Breast cancer is the most common malignancy in women worldwide and a molecularly heterogeneous disease (Harbeck et al., 2019; Siegel et al., 2019). The global incidence of breast cancer has been increasing from 1980 with an annual increase rate of 3.1%, and about 2.1 million women were newly diagnosed with breast cancer in 2018 (Bray et al., 2015, 2018). Current systematic therapies include chemotherapy, endocrine therapy (for hormone receptor-positive patients), anti-HER2 therapy (for HER2-positive patients), bone stabilizing agents, poly (ADP-ribose) polymerase inhibitors (for patients carrying BRCA mutation), and immunotherapy. Despite of improvement on these therapies, the mortality of breast cancer is still at a high level ranking the fourth among all cancer types worldwide (Bray et al., 2018). Therefore, it’s imperative to screen for novel and efficient therapeutic targets based on individual hereditary features.

Multiple molecular alterations have been reported, such as genomic mutation, transcriptomic and epigenetic dysregulation (Koboldt et al., 2012). Among these, microRNAs (miRNAs), which are small non-coding RNAs (∼22nt), have been shown to be aberrantly expressed and influence progression of breast cancer (Andorfer et al., 2011; Dong et al., 2020). More importantly, miRNAs have great potential to serve as effective markers for prognosis and therapies. A large number of studies have explored the important roles of miRNAs in human cancers, among them network analysis showed high efficiency to discover key miRNAs in cancer progression (Li et al., 2013; Bai et al., 2014; Shao et al., 2015; Suzuki et al., 2017; Chen et al., 2018). Li et al. (2013) constructed a functional miRNA-mRNA regulatory network (FMRN) associated with glioma malignant progression by using miRNA and mRNA profiling in 160 glioma patients with different tumor grades. Zhong et al. (2014) systematically analyzed miRNA-mRNA paired variations (MMPVs) and demonstrated widespread MMPVs in breast cancer. By an integrative analysis of miRNA-super enhancer network, Suzuki et al. (2017) found that super-enhancers drive the biogenesis of master miRNAs. Super-enhancers mark multiple miRNAs associated with cancer hallmarks, and the alterations of super-enhancers connected with multiple miRNAs were associated with cancer. Through network analysis, they identified keys miRNAs that regulate glioma malignant progression and predict survival of patient with glioma. However, most of miRNA dysregulated networks in previous studies were based on the dysregulation of miRNAs and target genes expression in cancer. In these dysregulated networks, the regulatory relations between miRNA and target genes were no evidently changed between cancer and normal samples. Analysis of dysregulated miRNA networks based on the dysregulation of regulatory relations between miRNAs and target genes may provide new insights for the important roles of miRNAs in breast cancer.

The Cancer Genome Atlas (TCGA) project has release unprecedented amount of molecular profiles in human cancers, which provided us great opportunity to reveal comprehensive molecular alterations of cancer, including DNA, RNA, protein, and epigenetic levels (Weinstein et al., 2013). Here, we constructed a dysregulated miRNA targeting network (DMTN) based the comparison of miRNA-mRNA regulation between breast cancer and normal breast samples derived from TCGA BRCA cohort. Our DMTN showed similarity to common biological networks. We applied SVM classifier on the miRNAs in DMTN and predicted 90 breast cancer risk miRNAs. Our further analysis showed that predicted miRNAs were important and participated in cancer and immune-related processes in breast cancer. More importantly, these risk miRNAs were associated with immune infiltration in breast cancer samples. Risk miRNAs were also found to be associated with many anti-cancer drugs across multiple cancer cell lines.

## Materials and Methods

### RNA-seq Expression Data

The expression profile of mRNA and miRNA used in this study were retrieved from the Genomic Data Commons data portal (GDC^1^). We obtained expression profile of 1,183 samples, including 1,079 invasive breast carcinoma and 104 adjacent normal samples. mRNA or miRNA with expression of zero in more than 10% of normal sample were removed as described in previous studies (Pan et al., 2018; Dong et al., 2020). Finally, 19,951 mRNAs and 1,881 miRNAs were filtered out for downstream analysis.

### Calculation of Dysregulation Scores

We obtained more than 2,500,000 miRNA-mRNA pairs from the Encyclopedia of RNA Interactomes (ENCORI) database (starBase v3.0^2^) (Li et al., 2014). These high-confidence miRNA-mRNA pairs were supported by AGO 1/2 crosslinking and immunoprecipitation (CLIP-seq) data. For each miRNA-mRNA pair, the dysregulation score was calculated as following:

S=|PCC(ijt)-PCC(ijn)|

Where *S* is the dysregulation score of miRNA *i* and mRNA *j*; *PCC(ijt)* denotes the Spearman correlation coefficient across tumor samples; *PCC(ijn)* represents the Spearman correlation coefficient across normal samples. Then sample labels were permutated and new dysregulation scores were calculated. The permutation was performed 10,000 times. The percent of times that permutated dysregulation scores were larger than observed ones was calculated as the *P* value. The dysregulation scores with *P* value < 0.05 were regarded as significant.

### Curation of Key Genes

For cancer-related gene, two resources were combined. One is compiled by Mertins et al. (2016) they manually collected 415 oncogenes and tumor suppressor genes from Uniprot database^3^ and published literatures. The other one comprises 524 cancer-related genes that harbor somatic mutations driving malignant transformation (Uhlén et al., 2015). In total, 724 cancer-related genes were collected. We also collected 5,619 housekeeping genes from a previous study (Jiang et al., 2016).

### Statistical Tests for the Significance of Overlap Between Two Sets

Predicted risk miRNAs and their one-step neighbor genes were supposed to be important miRNAs and genes in breast cancer. To explore the importance of predicted miRNAs, we overlapped these miRNAs with differential miRNA in breast cancer, cancer genes and housekeeping genes. The significance of overlap was calculated by a hypergeometric test as following:

$$\text{P}=1-\sum_{t=0}^{x}\frac{\left(\begin{array}{c} K\\ t \end{array}\right)\left(\begin{array}{c} N-M\\ M-t \end{array}\right)}{\left(\begin{array}{c} N\\ M \end{array}\right)}$$

Where *N* is the total number of miRNAs or genes expressed in TCGA BRCA cohort; *K* indicates the number of miRNA or gene set under investigation; and *M* denotes the number of interesting miRNAs or genes. In particular, the investigated sets were differential miRNAs in TCGA BRCA cohort, differentially expressed genes in TCGA BRCA cohort, cancer-related genes and housekeeping genes, respectively.

### Functional Enrichment Analysis

To investigate the possible biological functions that the dysregulated miRNA-mRNA network influence, functional enrichment analysis was performed by using Database for Annotation, Visualization and Integrated Discovery (DAVID v6.8) web server (Huang et al., 2009a, b). Specifically, one-step neighbor protein-coding genes of dysregulated miRNAs were subject to the Gene Ontology (GO) biological processes (Carbon et al., 2019) and Kyoto Encyclopedia of Genes and Genomes (KEGG) pathways (Kanehisa et al., 2017) to perform enrichment analysis. In addition, the TAM (version 2.0) (Li et al., 2018) web server was used to functionally annotate dysregulated miRNAs and then was subject to perform enrichment analysis.

### Survival Analysis

The clinical records of all breast cancer patients used in this study were also retrieved from GDC. Customized Perl scripts were used to extract regarding days to follow up and vital status of corresponding patients. For each miRNA or gene, the samples were divided into two groups by using median expression value as cutoff. Then the survival durations between different sample groups were compared using log-rank test. The related statistical test was implemented by using “survival” package.

### Evaluation of Immune Cell Infiltration in Breast Cancer Samples

In the present study, the CIBERSORT (Newman et al., 2015) method was adopted to estimate the relative abundance of different immune cell types in each patient sample. In particular, the predefined LM22 signature of immune cells in CIBERSORT algorithm was employed for immune cell deconvolution from gene expression. The LM22 immune cell signature is a leukocyte gene signature which comprises 547 genes that are able to distinguish 22 types of hematopoietic cells.

### Estimation of Association Between miRNAs/Genes and Anti-cancer Drugs

We first retrieved IC50 values of 266 anti-cancer drugs across 1,065 cancer cell lines from the Genomics of Drug Sensitivity in Cancer (GDSC) data portal (Yang et al., 2013), as well as miRNA/gene expression profiles of 1,019 cancer cell lines from the Cancer Cell Line Encyclopedia (CCLE) database. Cancer cell lines that have both expression and pharmacological profiles were kept for analysis. For each risk miRNA or one-step neighbor gene, Pearson correlation were computed across cell lines. The miRNA/gene-drug pairs with | R_*s*_| ≥ 0.2 and Benjamini-Hochberg adjusted *P* value < 0.05 were considered as statistically significant.

### Statistical Analysis and Plots

All statistical analysis and plots in the present study were performed in R environment^4^. Unless specific statement, a statistical test with *P* value or False Discovery Rate (FDR) < 0.05 was considered as significant.

## Results

### Construction of Dysregulated miRNA-mRNA Network in Breast Cancer

**FIGURE 1:**
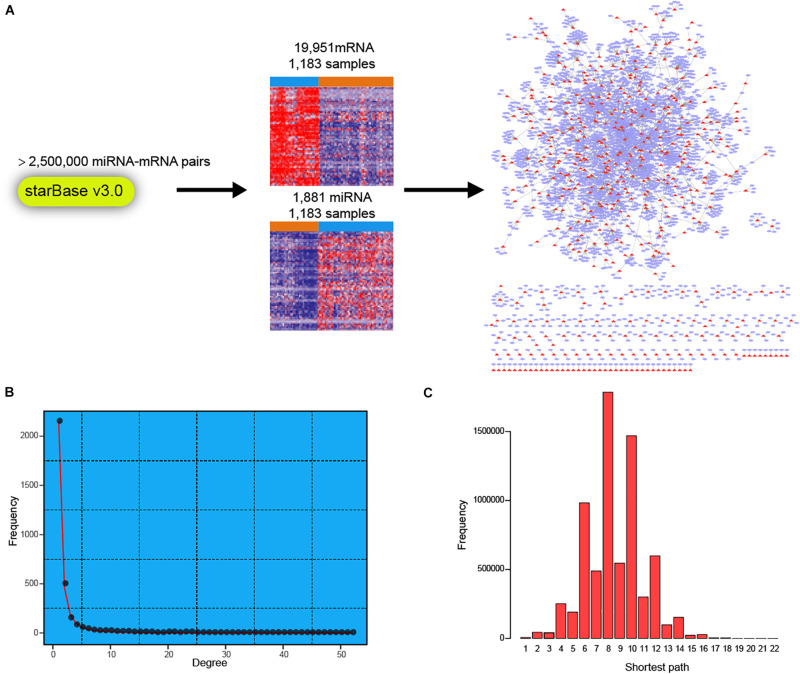
Construction of dysregulated miRNA-mRNA network in breast cancer. **(A)** Workflow of building the dysregulated miRNA-mRNA network in breast cancer. **(B)** The frequency distribution of degree in the network. **(C)** The frequency distribution of shortest path in the dysregulated miRNA-mRNA network.

To facilitate a deeper understanding of miRNA regulatory network in breast cancer, we first retrieved a high-confidence catalog of miRNA-mRNA pairs from starBase v3.0 (see section “Materials and Methods”). The miRNAs and mRNAs were then used to extract corresponding expression profiles in TCGA BRCA cohort (Figure 1A). Finally, expression profiles of 1,183 miRNAs and 19,951 mRNAs across 1,183 breast cancer samples were obtained for miRNA-mRNA network construction. Briefly, the expression correlations between miRNAs and corresponding mRNAs were calculated in normal breast and breast cancer samples, respectively. Then, the miRNA targeted dysregulated network (MTDN) in breast cancer was constructed from miRNA-mRNA pairs with significant dysregulation scores (see section “Materials and Methods”). The out-degree of miRNAs, which was defined as the number of outgoing edges per node, was computed in the MTDN. We found that miRNA out-degree showed a power-law distribution, wherein most miRNAs were found to be lowly connected and a few was relatively highly connected (Figure 1B). In addition, the shortest paths also exhibited a biological network distribution with most miRNAs ranging from 6 to 10 (Figure 1C and Supplementary Figure S1). These results indicate that our MTDN was not random but similar to most biological network, with a core set of miRNAs. Analysis based on this MTDN will lead to the discovery of key regulators in breast cancer.

### Prediction of Risk miRNAs by SVM Classifier

**FIGURE 2:**
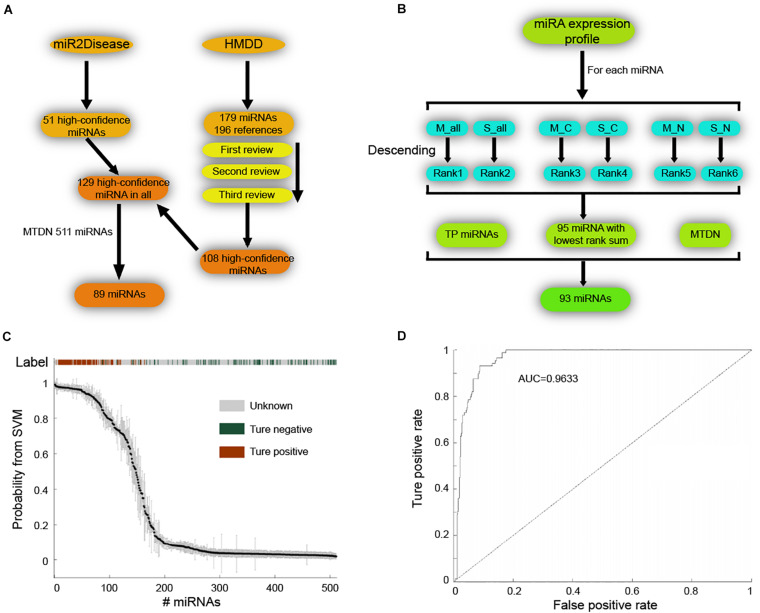
Establishing SVM classifier for dysregulated miRNAs in breast cancer. **(A)** Workflow of retrieving true positive miRNA set. **(B)** Procedure of obtaining true negative miRNA set for training SVM classifier. **(C)** The SVM classification of samples with different labels, including true positive, true negative and unknown. **(D)** AUC curve (false positive rate V.S. true positive rate) of SVM classifier performance.

Next, we applied SVM machine learning on the MTDN to infer breast cancer risk miRNAs. The true positive (TP) and true negative (TN) miRNA sets were collected to train the SVM classifier. For the TP set of miRNAs, 51 high-confidence miRNAs and 179 miRNAs were retrieved from the miR2Disease (Jiang et al., 2009) and HMDD (Huang et al., 2019) databases, respectively (Figure 2A). The 179 miRNAs of HMDD were collected from 196 references and were manually reviewed three times. These two sets of miRNAs were merged into a set of 129 unique high-confidence miRNAs. Finally, 89 TP miRNAs were obtained by overlapping these 129 miRNAs with 511 miRNAs in MTDN. To get the TN miRNAs, we first separately ranked the miRNAs in all samples, breast cancer samples and normal breast samples based on the average expression values and standard variance values across samples, separately (Figure 2B). In each sample type, two ranks were used, e.g., median values and standard variance values. Then, 95 miRNAs with the lowest sum of ranks were selected, which means these miRNAs have lowest expression values and lowest variation across samples, implying the functionless roles of these miRNAs. These 95 miRNAs were further filtered by excluding miRNAs that were involved in the TP miRNA set, and were not involved in the MTDN. Finally, we got 93 miRNAs in the TN set. The difference of five features (i.e., out-degree in MTDN, the number of co-regulating miRNAs, the percentage of co-regulating disease miRNAs, the number of genes regulated by disease miRNAs, and expression fold change of mean values in breast cancer vs. normal samples.) between TP and TN was further evaluated. Each feature we selected is significantly different between TP and TN sets, which indicates that our features are sufficient to train the SVM classifier (Supplementary Figure S2). The selected TP and TN miRNA sets and five features were employed to train the SVM classifier by using five-fold cross validation. Genetic algorithm (GA) was used to choose the optimal parameters in the SVM classifier. The recognition rate was set as 0.825, because the SVM classifier identifies no TN miRNAs when the recognition rate is larger than 0.825 (Figure 2C). The trained SVM classifier obtained an AUV of 0.9633 (Figure 2D). These observations demonstrate the strong predicting of trained SVM classifier from our MTDN.

### Predicted miRNAs Show High Importance in Breast Cancer

**FIGURE 3:**
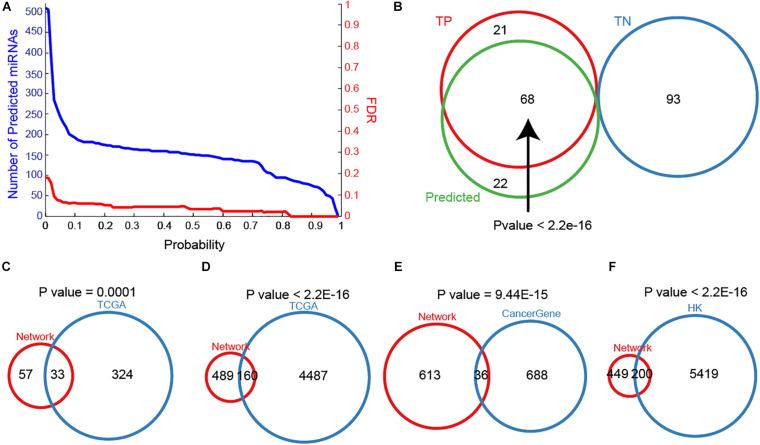
The importance of predicted miRNAs. **(A)** The distribution of miRNA numbers and significance (FDR) with different predicted probabilities. **(B)** The overlap between true positive, true negative and predicted samples. Overlaps between sets of predicting miRNAs and differential miRNAs in TCGA BRCA cohort **(C)**, one-step neighbors of predicted miRNAs and differential protein-coding genes in TCGA BRCA cohort **(D)**, **(E)** one-step neighbors of predicting miRNAs and cancer-associated genes, and one-step neighbors of predicting miRNAs and housekeeping genes **(F)**.

To maximize the utility of MTDN in breast cancer, we further integrated the SVM classifier with MTDN. Trained SVM classifier was applied on 511 miRNAs in MTDN and 90 miRNAs were predicted to be breast cancer risk miRNAs (Figure 3A). Among the identified 90 risk miRNAs, 68 were in the TP miRNA set and 22 were novel risk miRNAs (Figure 3B). Notably, no predicted risk miRNAs were included in the TN miRNA set, demonstrating strong predictability (*P* < 2.2E-16) and stability of the trained SVM classifier based on MTDN. In addition, about one third (33 in 99) of the predicted risk miRNAs were overlapping with differential miRNAs in TCGA BRCA cohort (*P* = 0.0001, Hypergeometric test) (Figure 3C). As expected, the one-step neighbor genes of predicted risk miRNAs were significantly involved in differential genes of TCGA BRCA cohort with 160 differential genes (24.65%, *P* < 2.2E-16, Hypergeometric test) (Figure 3D). Furthermore, 36 of 649 one-step neighbor genes were cancer-related genes (*P* = 9.44E-15, Hypergeometric test) (Figure 3E). And 200 of risk miRNA one-step neighbor genes were housekeeping (HK) genes (*P* < 2.2E-16, Hypergeometric test) (Figure 3F). Our observations demonstrated that risk miRNAs predicted from MTDN were important miRNAs in breast cancer.

### Predicted Risk miRNAs Were Biologically and Clinically Relevant in Breast Cancer

**FIGURE 4:**
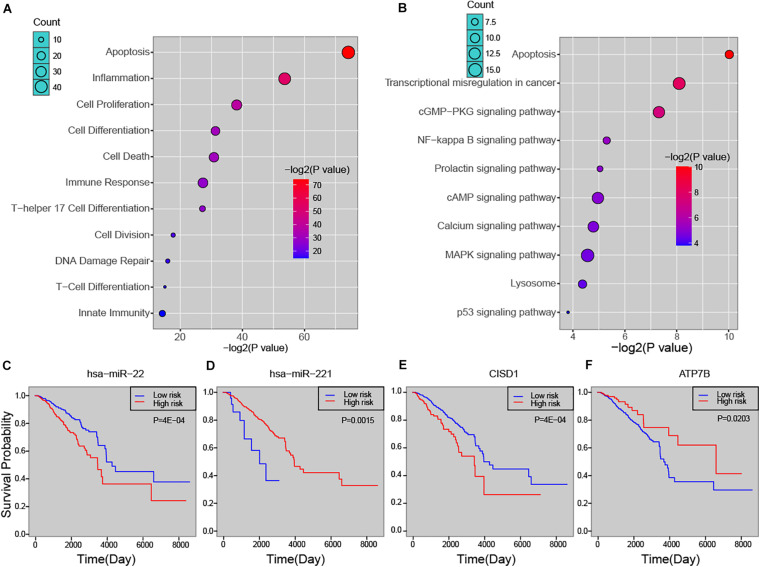
The biological and clinical relevance of predicted miRNAs. **(A)** Functional enrichment analysis of predicted miRNAs. **(B)** Enriched pathways of one-step neighbors of predicted miRNAs. Kaplan-Meier survival curve of hsa-miR-22 **(C)**, hsa-miR-221 **(D)**, CISD1 **(E)**, and ATP7B **(F)**.

In order to explore the biological process that the risk miRNA may affect in the progression of breast cancer, we performed functional enrichment analysis. By investigating miRNA-function set annotated in the TAM 2.0 database, we first performed enrichment analysis by using miRNA annotated biological functions (see section “Materials and Methods”). In addition to basic cancer biological processes, predicted risk miRNAs were found to be enriched in several immune-related functions, such as “lymphocyte activation,” “T cell differentiation,” and “leukocyte differentiation” (Supplementary Figure S3A). The analysis of ECGAP SAGE showed that risk miRNAs were highly associated with breast cancer-related terms, such as “mammary gland invasive breast cancer ER^+^, PR^+^, HER2^–^, grade II” and “mammary gland normal breast tissue from a breast cancer patient” (Supplementary Figure S3B). Furthermore, one-step neighbor genes of risk miRNAs were subject to enrichment analysis. We found that these genes were significantly enriched in principal cancer-related functions, such as “Apoptosis,” “Cell Proliferation,” “DNA Damage Repair” and immune-related processed, such as “Inflammation,” “Immune Response,” “T-Cell Differentiation” (Figure 4A). Key cancer-related pathways were also significantly enriched by one-step neighbor genes, such as “MAPK signaling pathway,” “cAMP signaling pathway” and “NF-kappa B signaling pathway” (Figure 4B). We further evaluated the association of risk miRNAs with patient survival time. Predicted risk miRNAs and one-step neighbor genes were found to be significantly associated with survival time of breast cancer patients (see section “Materials and Methods”). For example, high expression of hsa-miR-22 was significantly associated with short survival time (*P* = 4E-04, log-rank test) (Figure 4C), and higher expression of hsa-miR-221 indicated a better outcome of breast cancer patients (*P* = 0.0015, log-rank test) (Figure 4D). Higher expression level of CISD1 was found to be associated with worse clinical outcome (*P* = 4E-04, log-rank test) (Figure 4E), and high expression of ATP7B was significantly associated with long survival time of breast cancer patients (*P* = 0.0203, log-rank test) (Figure 4F). Besides, multiple other risk miRNAs and one-step neighbor genes showed significant association with patient survival time, such as hsa-miR-195 (*P* = 0.0061), hsa-miR-214 (*P* = 0.0061), hsa-miR-328 (*P* = 0.0149), BCR (*P* = 0.0235), C2orf72 (*P* = 0.0112) and CLBA1 (*P* = 0.0278) (Supplementary Figure S4). Our analysis showed that predicted risk miRNAs were biologically and clinically relevant in breast cancer.

### Risk miRNAs Were Associated With Immune Cell Infiltration in Breast Cancer

**FIGURE 5:**
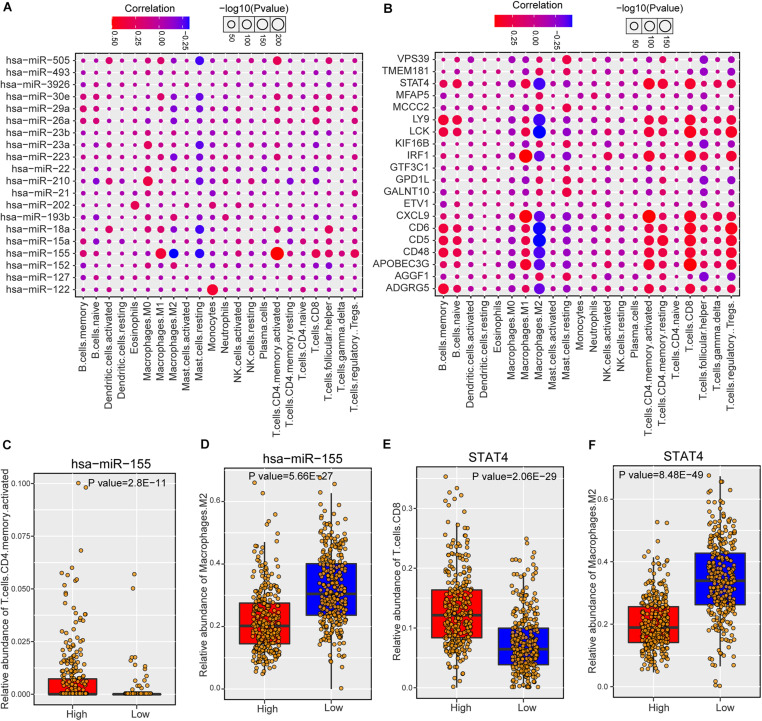
The association between predicted miRNAs and immune cell infiltration of breast cancer. **(A)** The correlations between predicted miRNAs (top 20) and immune cell infiltrations. **(B)** The associations between one-step neighbors (top 20) of predicted miRNAs and immune cell infiltrations. **(C)** Comparison of CD4 memory activated T cells abundance between hsa-miR-155 high and low samples. **(D)** Comparison of M2 macrophages abundance between hsa-miR-155 high and low samples. **(E)** Comparison of CD8 T cells between STAT4 high and low samples. **(F)** Comparison of M2 macrophages abundance between STAT4 high and low samples.

As risk miRNAs were found to be involved in some immune-related functions, we further investigate the possible roles of risk miRNAs in immunology of breast cancer. We estimated the associations between risk miRNA expression levels and immune cell infiltration across breast cancer samples. The relative abundance of 22 different immune cell types in each sample was first calculated by using CIBERSORT algorithm (see section “Materials and Methods”). Then the Spearman correlation between expression level of each miRNA and abundance of each immune cell type was calculated to infer their association. In addition, the relations between one-step neighbors of risk miRNAs and immune cell infiltration were also evaluated. The majority of risk miRNAs were highly associated with immune cell infiltrations of breast cancer samples (Supplementary Figure S5A). As expected, one-step neighbor genes also showed remarkable association with immune cells (Supplementary Figure S5B). We showed the detailed association of top 20 risk miRNAs in Figure 5A and top 20 one-step neighbor genes in Figure 5B. Most of the immune cells were positively associated with risk miRNAs and one-step neighbor gene, while a small portion showed negative association. For example, resting mast cells showed consistently negative association with risk miRNAs and M2 macrophages were negatively associated with most one-step neighbor genes. We found that high expression of hsa-miR-155 indicated high infiltration level of activated CD4+ T cells but low abundance of M2 macrophages (Figures 5C,D). Additionally, the high expression level of STAT4 gene denoted high abundance of CD8+ T cells but low infiltration level of M2 macrophages (Figures 5E,F). Our analysis suggested that risk miRNAs may participate in the regulation of immune cells, shedding lights on the possibility of targeting miRNAs in immunotherapy for patients with breast cancer.

### Risk miRNAs Show High Association With Anti-cancer Drugs Across Cancer Cell Lines

**FIGURE 6:**
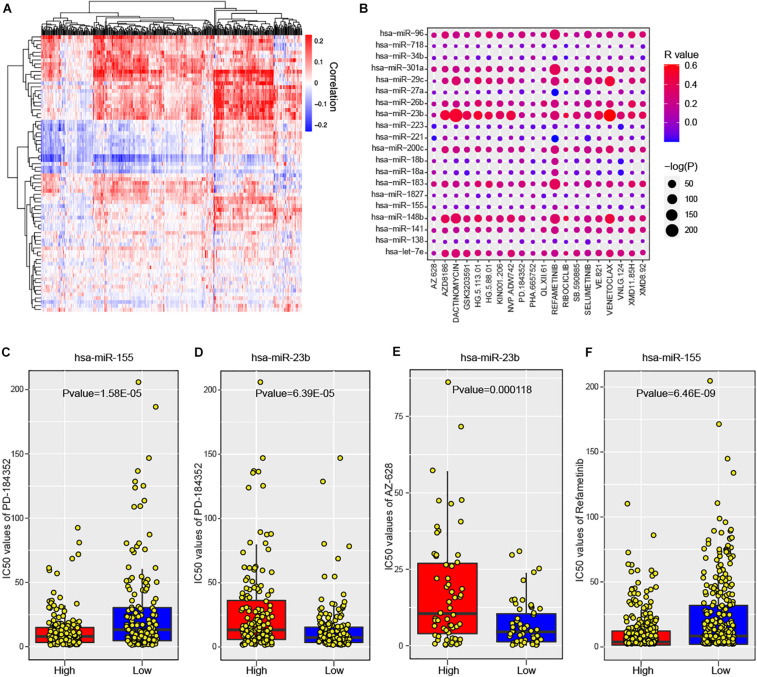
The association between predicted miRNAs and anti-cancer drugs across human cancer cell lines. **(A)** Heatmap shows the significant correlations between predicted miRNAs and anti-cancer agents. **(B)** The significance of associations between top 20 miRNAs and anti-cancer drugs. **(C)** Comparison of cell response to PD-184352 (IC50 values) between hsa-miR-155 high and low cell lines. **(D)** Comparison of cell response to AZ-628 (IC50 values) between hsa-miR-23b high and low cell lines. **(E)** Comparison of cell response to PD-184352 (IC50 values) between hsa-miR-23b high and low cell lines. **(F)** Comparison of cell response to Refametinib (IC50 values) between hsa-miR-155 high and low cell lines.

To maximize the clinical utility of predicted risk miRNAs, we further investigated the correlations between the expression levels of risk miRNAs and 266 anti-cancer drugs across 1,065 cancer cell lines (see section “Materials and Methods”). The majority of miRNAs showed consistent response to most anti-cancer drugs (Figure 6A). In particular, 588 significant miRNA-drug pairs between 50 miRNAs and 222 drugs were identified, wherein most associations were positive (580, 98.6%). For one-step neighbor genes, 3,146 significant gene-drug pairs were identified (Supplementary Figure 6A). The top 20 miRNA-drug association was shown in Figure 6B and top 20 gene-drug association was displayed in Supplementary Figure 6B. These results indicated that the expression levels of risk miRNAs and one-step neighbor genes could be used to infer the sensitivity of cell lines to anti-cancer drugs. For example, cancer cell lines with high level of hsa-miR-155 were more sensitive to PD-184352 (Figure 6C, *P* value = 1.58E-05), while higher expression of hsa-miR-23b indicated more resistant to PD-184352 (Figure 6D, *P* value = 6.39E-05). High expression of hsa-miR-23b indicated significantly higher resistance of cancer cell lines to AZ-628 (Figure 6E, *P* value = 0.000118). Cancer cell lines with high expression levels of hsa-miR-155 showed more sensitive response to Refametinib (Figure 6F, *P* value = 6.46E-09). Furthermore, one-step neighbor genes also showed the potential to indicate the response of cancer cells to anti-cancer drugs. For example, the expression level of MTF2 was significantly positively associated with sensitivity to GSK429286A (Supplementary Figure S6C, *P* value = 1.21E-06). Low expression of RNF138 was significantly associated with resistance of cancer cells to Quizartinib (Supplementary Figure S6D, *P* value = 3.03E-11). High expression of MTF2 was also found to be associated with the sensitivity of Zibotentan across cancer cell lines (Supplementary Figure S6E, *P* value = 5.93E-09). Cancer cell lines with high expression of MTF2 were more resistant to Refametinib (Supplementary Figure S6F, *P* value = 6.89E-10). In summary, our analysis suggested that risk miRNAs and their one-step neighbor genes could be used as indicators of the sensitivity of cancer cell lines to anti-cancer drugs.

## Discussion

The importance of miRNAs in human cancers has been proven in many studies. But most of previous studies were based on the expression dysregulation of miRNAs and target genes. In this study, we constructed dysregulated miRNA target network (DMTN) based on the dysregulation of regulatory relations between miRNA and target genes. Our proposed DMTN show similarity with common biological networks. We identified important breast cancer miRNAs in this DMTN. These breast cancer risk miRNAs were found to be involved in many cancer-related and immune-related processes. These results demonstrated that our DMTN was biologically meaningful and could be applied to other cancer types.

In the training of SVM classifier, we only used miRNA and mRNA expression profiling from TCGA BRCA cohort. To our knowledge, this is the largest breast cancer cohort with clinical information up to date. Validation of our SVM classifier in other breast cancer datasets may improve the predicting power. But we already got a very high AUC (0.9633), the space for improvement is very small.

Interestingly, we found that predicted risk miRNAs and one-step neighbor genes were significantly associated with immune cell infiltration in breast cancer samples. We used CIBERSORT algorithm to infer the relative infiltration levels of different immune cells in breast cancer samples. Although predicting cell infiltration from mixed samples may be distracted by many factors, the computational method we used could provide a relatively accurate prediction. We believe that the majority of association between miRNA expression and immune cell abundance could reflect the real association. These findings could be validated by using single cell sequencing and other biological technologies. Our findings provide new insights of miRNA roles in breast cancer and shed lights on immunotherapy for patients with breast cancer.

Furthermore, we identified 588 miRNA-drug and 3,146 gene-drug pairs that miRNAs/genes expression were significantly associated with the response of cancer cells to anti-cancer drugs. Our results suggested that the expression levels of risk miRNAs and their one-step neighbor genes could be indicators for the sensitivity of cancer cells to anti-cancer drugs. With further experimental and clinical validations, these miRNAs could be used as biomarkers guiding treatment of patients with breast cancer. Our study will promote the precision medicine for breast cancer patients.

## Data Availability Statement

The miRNA, mRNA expression and clinical data can be found at the GDC data portal (https://portal.gdc.cancer.gov/). Software and resources used for analysis are described in each method section. All results generated in this study can be found in Supplementary Tables.

## Author Contributions

DL and CJ conceived the project. MQ, QF, and CJ collected the data. MQ performed bioinformatics analysis and wrote the manuscript. MQ, QF, CJ, and DL reviewed the manuscript. All authors contributed to the article and approved the submitted version.

## Conflict of Interest

The authors declare that the research was conducted in the absence of any commercial or financial relationships that could be construed as a potential conflict of interest.

## Footnotes

1 https://portal.gdc.cancer.gov/

2 http://starbase.sysu.edu.cn/index.php

3 https://www.uniprot.org/

4 https://www.r-project.org/
