# Supplementary material for: Machine Learning Based Network Analysis Determined Clinically Relevant miRNAs in Breast Cancer
Source: Front Genet. 2020 Nov 12;11:615864. doi: 10.3389/fgene.2020.615864 (PMC7689188; doi:10.3389/fgene.2020.615864)
Supplement: Supplementary file 1 [file Data_Sheet_1.PDF]

Supplementary Figure S1

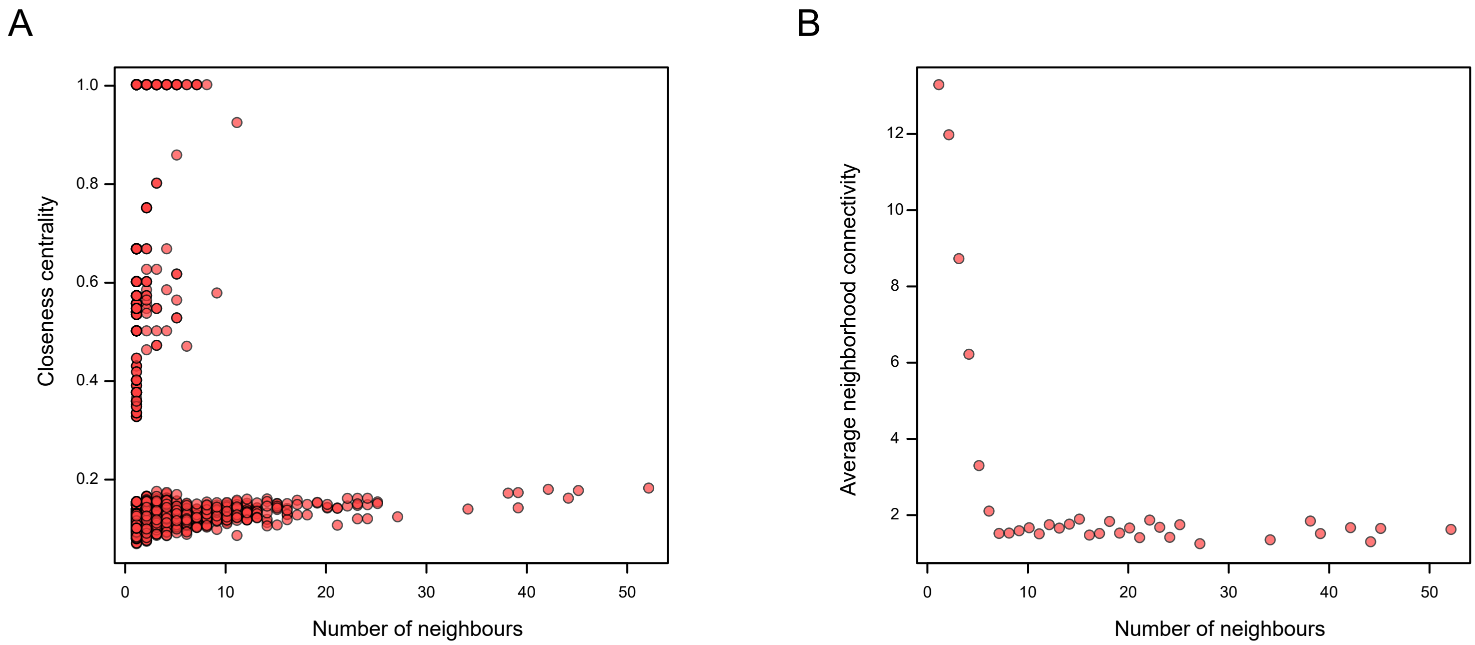

Supplementary Figure S1. Topological property of dysregulated miRNA-mRNA network in breast cancer. (A) Distribution of closeness centrality based on neighbor numbers. **(B)** The distribution of average neighborhood connectivity based on the number of neighbors.

Supplementary Figure S2

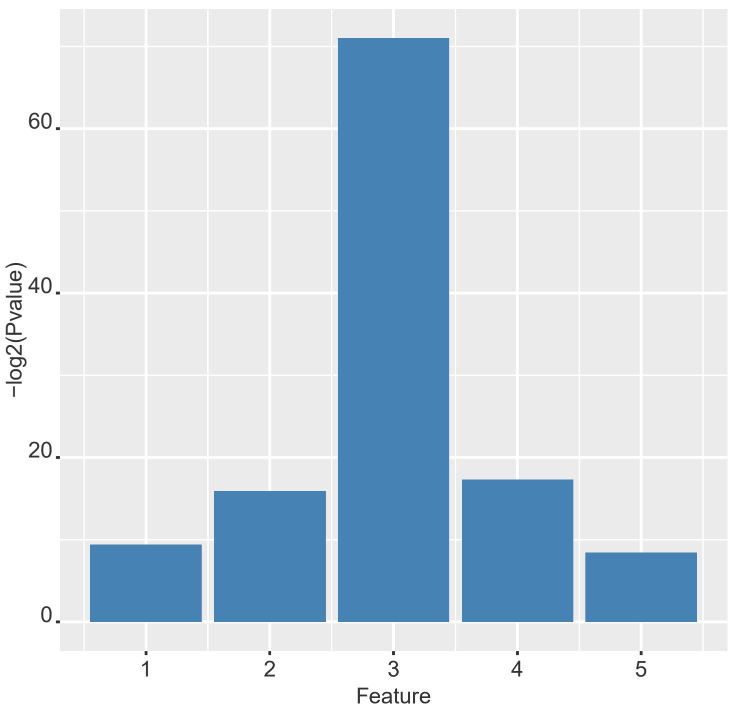

Supplementary Figure S2. Difference of each feature between TP and TN sets

Supplementary Figure S3

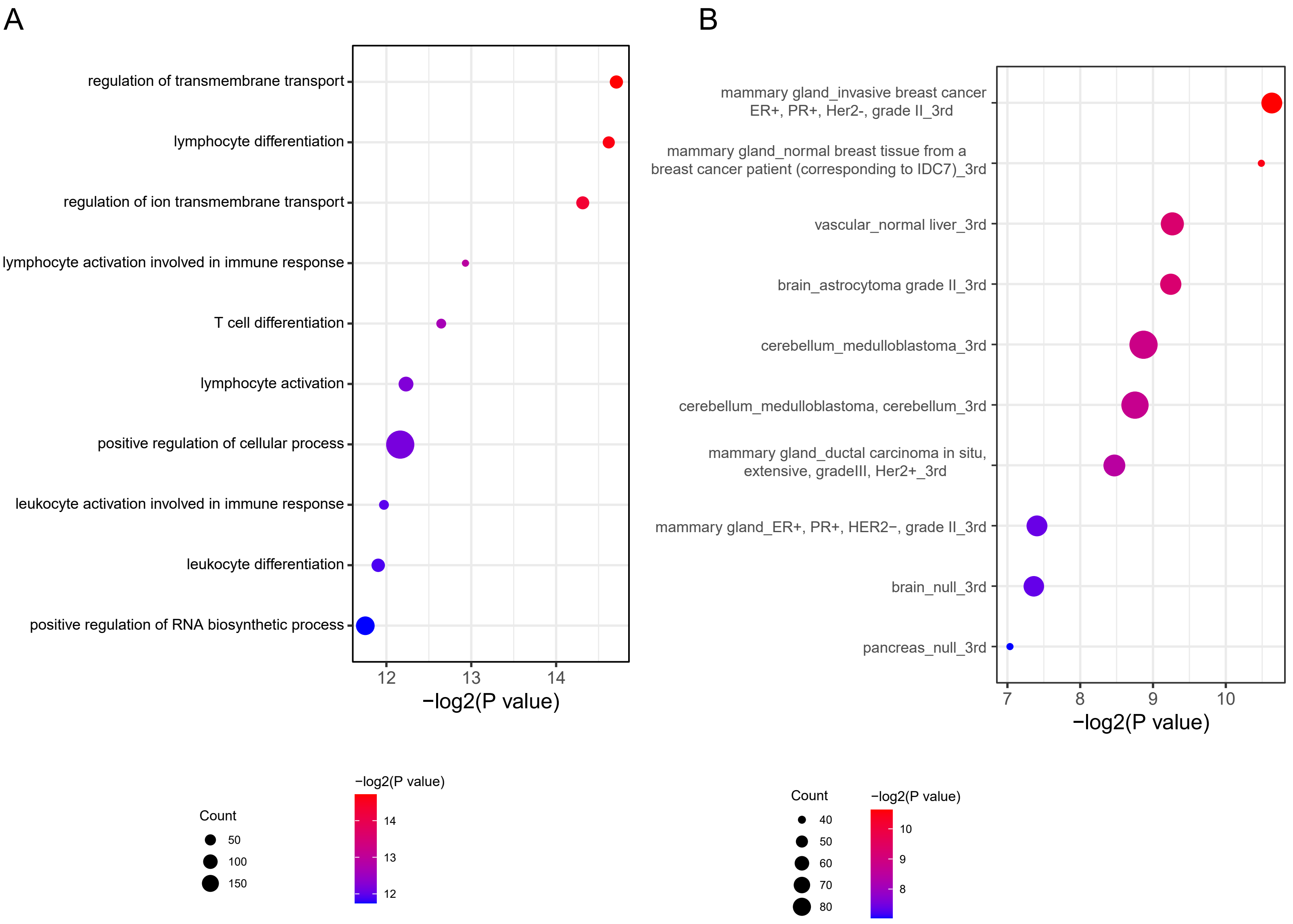

Supplementary Figure S3. Enriched biological processes of predicted risk miRNAs.

Supplementary Figure S4

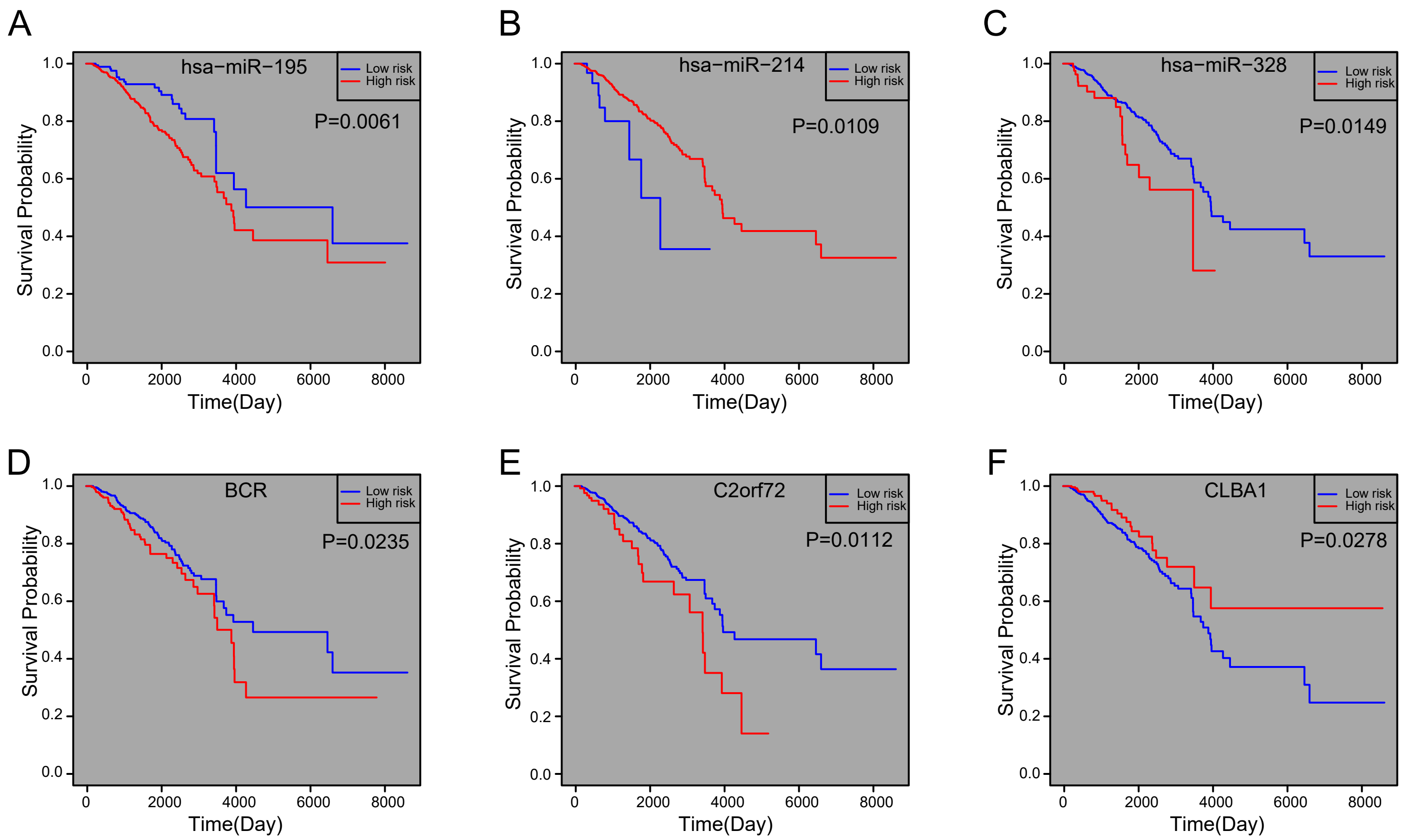

Supplementary Figure S4. Kaplan-Meier survival curves of hsa-miR-195 (**A**), hsa-miR-214 (**B**), hsa-miR-328 (**C**), BCR (**D**), C2orf72 (**E**), and CLBA1 (**F**).

Supplementary Figure S5

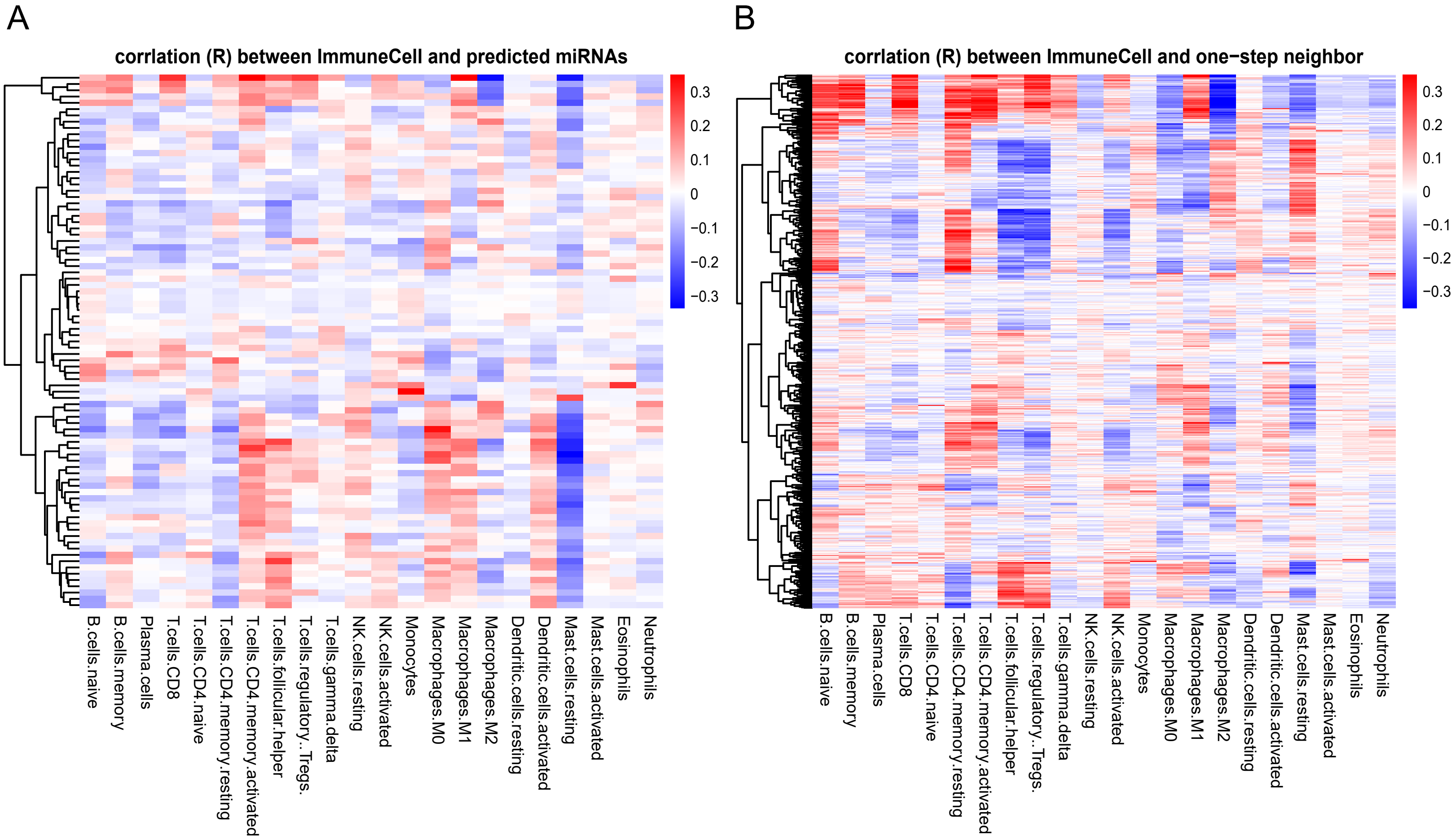

Supplementary Figure S5. The assoication of risk miRNAs and immune cell infiltration in breast cancer. (A) Heatmap shows the correlations between risk miRNAs and infiltrations of different immune cells. **(B)** Heatmap shows the associations between one-step neighbors of risk miRNAs and immune cell infiltration.

Supplementary Figure S6

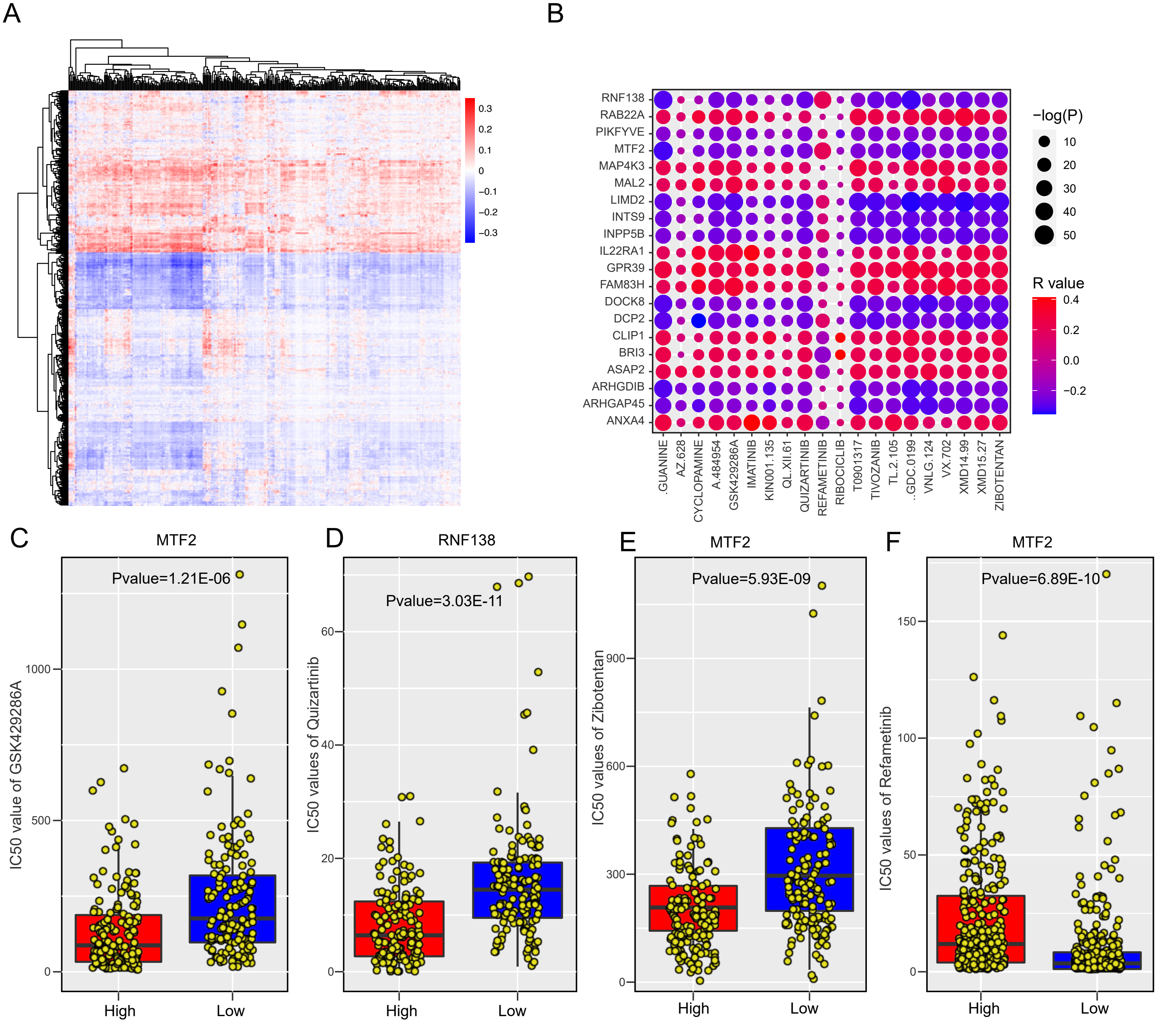

Supplementary Figure S6. The assoication of one-step genes and anti-cancer drugs across cancer cell lines. (A) Heatmap shows the correlations between one-step genes and anti-cancer drugs. **(B)** The significance of correlations between top 20 one-step genes and anti-caner drugs. (**C**) Comparison of cell response to GSK429286A between MTF2 high and low cell lines. (**D**) Comparison of cell response to Quizartinib between RNF138 high and low cell lines. (**E**) Comparison of cell response to Zibotentan between MTF2 high and low cell lines. (**F**) Comparson of cell response to Refametinib between MTF2 high and low cell lines.
